# Supplementary material for: Resolving the full spectrum of human genome variation using Linked-Reads
Source: Genome Res. 2019 Apr;29(4):635–45. doi: 10.1101/gr.234443.118 (PMC6442396; doi:10.1101/gr.234443.118)
Supplement: Supplemental Material [file supp_gr.234443.118_Supplemental_longranger-2.2.zip › longranger-2.2.tar.bz2!/lr2.2-public/lib/stan/cmdstan/stan/doc/api/html/check__spsd__matrix_8hpp_source.html]

Stan: src/stan/math/error\_handling/matrix/check\_spsd\_matrix.hpp Source File


|  |  |
| --- | --- |
|  | Stan  2.5.0  probability, sampling & optimization |


- Main Page
- Related Pages
- Namespaces
- Classes
- Files

- File List
- File Members

All Classes Namespaces Files Functions Variables Typedefs Enumerations Enumerator Friends Macros Pages

- src
- stan
- math
- error\_handling
- matrix

check\_spsd\_matrix.hpp

Go to the documentation of this file.

1 #ifndef STAN\_\_MATH\_\_ERROR\_HANDLING\_\_MATRIX\_\_CHECK\_SPSD\_MATRIX\_HPP

2 #define STAN\_\_MATH\_\_ERROR\_HANDLING\_\_MATRIX\_\_CHECK\_SPSD\_MATRIX\_HPP

3

4 #include <sstream>

5 #include <stan/math/matrix/Eigen.hpp>

6 #include <stan/math/error\_handling/check\_positive.hpp>

7 #include <stan/math/error\_handling/matrix/check\_pos\_semidefinite.hpp>

8 #include <stan/math/error\_handling/matrix/check\_symmetric.hpp>

9 #include <stan/math/error\_handling/matrix/check\_square.hpp>

10

11 namespace stan {

12  namespace math {

13

27  // FIXME: update warnings

28  template <typename T\_y, typename T\_result>

29  inline bool check\_spsd\_matrix(const char\* function,

30  const Eigen::Matrix<T\_y,Eigen::Dynamic,Eigen::Dynamic>& y,

31  const char\* name,

32  T\_result\* result) {

33  check\_square(function, y, name, result);

34  check\_positive(function, y.rows(), "rows", result);

35  check\_symmetric(function, y, name, result);

36  check\_pos\_semidefinite(function, y, name, result);

37  return true;

38  }

39

40  }

41 }

42 #endif

check\_positive.hpp

stan::math::check\_spsd\_matrix

bool check\_spsd\_matrix(const char \*function, const Eigen::Matrix< T\_y, Eigen::Dynamic, Eigen::Dynamic > &y, const char \*name, T\_result \*result)

Return true if the specified matrix is a square, symmetric, and positive semi-definite.

**Definition:** check\_spsd\_matrix.hpp:29

stan::math::check\_pos\_semidefinite

bool check\_pos\_semidefinite(const char \*function, const Eigen::Matrix< T\_y, Eigen::Dynamic, Eigen::Dynamic > &y, const char \*name, T\_result \*result)

Return true if the specified matrix is positive definite.

**Definition:** check\_pos\_semidefinite.hpp:33

check\_pos\_semidefinite.hpp

check\_symmetric.hpp

stan::math::check\_symmetric

bool check\_symmetric(const char \*function, const Eigen::Matrix< T\_y, Eigen::Dynamic, Eigen::Dynamic > &y, const char \*name, T\_result \*result)

Return true if the specified matrix is symmetric.

**Definition:** check\_symmetric.hpp:31

stan::math::check\_positive

bool check\_positive(const char \*function, const T\_y &y, const char \*name, T\_result \*result)

**Definition:** check\_positive.hpp:54

Eigen.hpp

stan::math::check\_square

bool check\_square(const char \*function, const Eigen::Matrix< T\_y, Eigen::Dynamic, Eigen::Dynamic > &y, const char \*name, T\_result \*result)

Return true if the specified matrix is square.

**Definition:** check\_square.hpp:24

check\_square.hpp


---

[ Stan Home Page ]

*© 2011–2014,
Stan Development Team.*
